# Dual functionality of MDM2 in PROTACs expands the horizons of targeted protein degradation

**DOI:** 10.1186/s40364-025-00826-7

**Published:** 2025-08-27

**Authors:** Junyi Zhao, Hongzhen Chen, Chao Liang

**Affiliations:** 1 Department of Systems Biology, School of Life Sciences, Southern University of Science and Technology, Shenzhen, 518055 China; 2 State Key Laboratory of Proteomics, National Center for Protein Sciences (Beijing), Beijing Institute of Lifeomics, Beijing, 100850 China

**Keywords:** TPD, PROTAC, MDM2, Bridged PROTAC, Homo-PROTAC

## Abstract

The evolution of targeted protein degradation (TPD) has been significantly propelled by the advent of proteolysis-targeting chimeras (PROTACs), which utilize heterobifunctional molecules to facilitate the ubiquitination-mediated degradation of previously “undruggable” proteins. Mouse double minute 2 (MDM2), which is often overexpressed in various diseases and plays a crucial role in regulating key pathways like p53, emerges as an exemplary candidate for therapeutic exploitation within the TPD realm, serving both as an intrinsic E3 ligase and as a direct protein of interest (POI). By harnessing MDM2’s inherent E3 ligase activity, PROTACs have been designed to efficiently degrade specific POIs, achieving substantial success in both in vitro and in vivo studies. Alternatively, PROTACs have been developed to directly target MDM2 itself, offering new approaches for therapeutic intervention. Recent research has yielded valuable strategies for optimizing MDM2-harnessing and MDM2-targeted PROTAC designs, concentrating on warhead selection of POI, linker length and composition optimization, and the choice among various E3 ligases and their corresponding recruiters. These advancements not only broaden the scope of PROTAC technologies but also expedite the development of MDM2-based therapies, inspiring approaches for disease treatment.

## Introduction

**Fig. 1:**
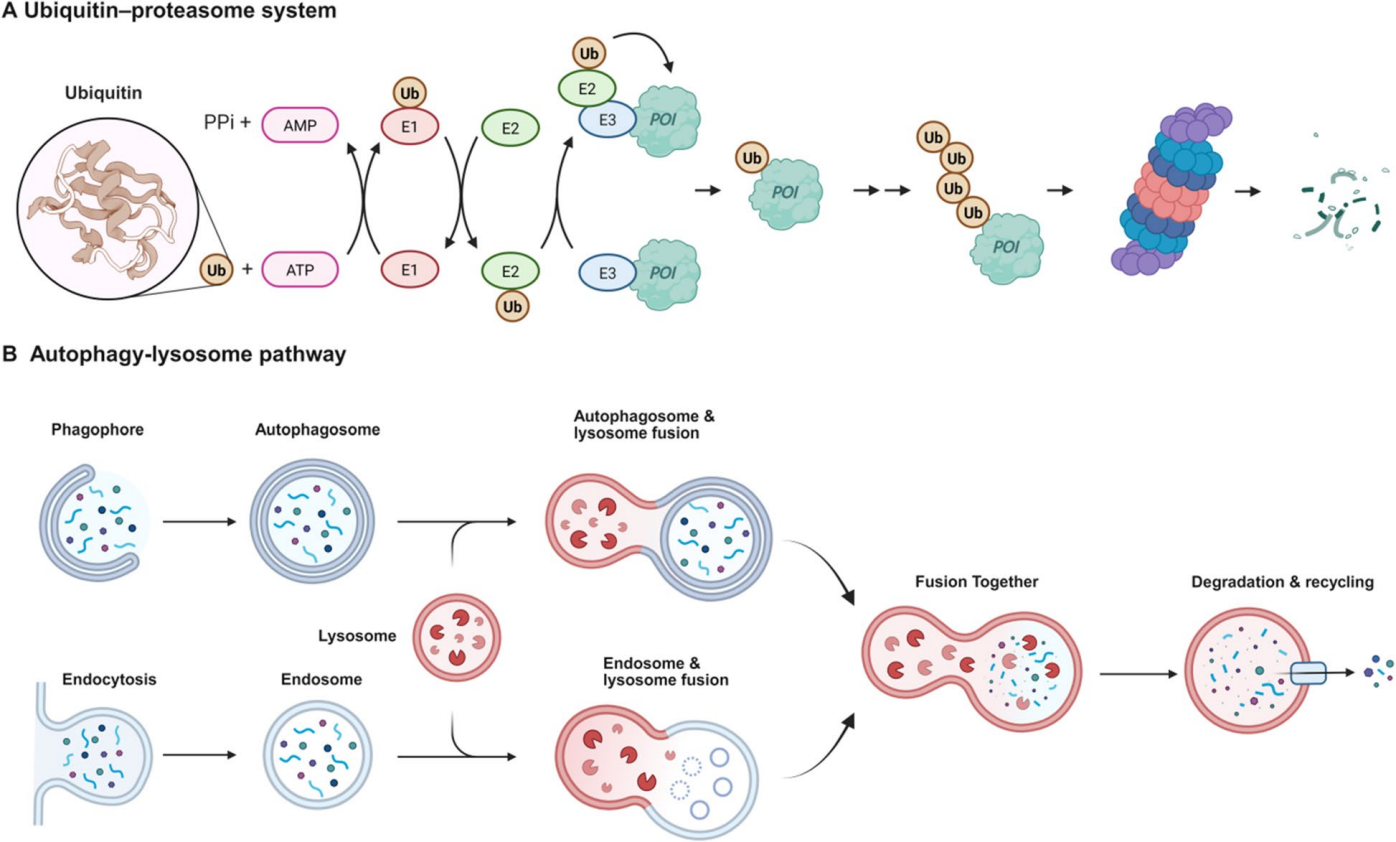
Two protein degradation pathways. **A** Ubiquitin–proteasome system. This system is the primary pathway for selective protein degradation in eukaryotic cells. It targets short-lived, misfolded, or damaged proteins for destruction in a tightly regulated process. The 26S proteasome, a barrel-shaped protease complex, recognizes ubiquitinated proteins, removes the ubiquitin tags via deubiquitinases, and unfolds and degrades substrates into short peptides. **B** Autophagy-lysosome pathway. Lysosomes are membrane-bound organelles containing hydrolytic enzymes that degrade macromolecules via autophagy or endocytosis. The key roles of lysosomes include the degradation of long-lived proteins, the clearance of aggregated proteins, and the recycling of organelles

Proteostasis refers to the homeostatic regulation of the proteome, and involves the coordinated control of protein synthesis, folding, trafficking, modification, and degradation [1–3]. Various diseases, including age-related neurodegenerative disorders, metabolic diseases, muscle-wasting conditions, and cancers, are characterized by failures in proteostasis [4–6]. The proteostasis network handles the entire lifecycle of proteins through cellular pathways, starting with synthesis and folding [2, 7]. It involves cytosolic and mitochondrial translation as well as chaperone-mediated folding. Chaperones use ATP-dependent and -independent mechanisms to prevent misfolding and aggregation, ensuring proper protein maturation [8–10]. Protein functionality is also influenced by post-translational modifications and subcellular location, with approximately two-thirds of proteins needing transport from their synthesis site in the cytosol to specific subcellular compartments [1, 11, 12]. Additionally, the precise regulation of protein abundance is vital for maintaining cellular homeostasis through ensuring proper cell signaling processes, optimal substrate turnover rates within metabolic pathways, and the correct assembly of complex macromolecular structures [1, 12]. Besides restricting protein synthesis, protein degradation via the ubiquitin–proteasome system (UPS) and the autophagy-lysosome pathway (ALP) plays a significant role in controlling protein abundance and eliminating dysfunctional proteins [13–16] (Fig. 1). Among the components of the proteostasis network, the number of unique genes of UPS (1286) and ALP (849) accounts for over two-thirds, which implies the importance of meticulous control of protein degradation [5, 6, 17, 18].

The UPS degrades around 80% to 90% of cellular proteins, especially up to 90% in mammals [19]. The process starts with the ubiquitin-activating enzyme (E1) using ATP to attach ubiquitin to its active site, forming a high-energy thioester bond [20]. The activated ubiquitin is then transferred to a ubiquitin-conjugating enzyme (E2) and finally to the target protein via a ubiquitin ligase (E3) [21, 22]. E3 enzymes are responsible for selectively recognizing appropriate substrate proteins through short peptide motifs, known as linear degrons, or through specific structural features, often referred to as structural degrons [23]. Multiple ubiquitin molecules are linked together to form a lysine-48 (K48)-linked polyubiquitin chain, which is recognized by the 26S proteasome. Subsequently, the proteasome translocates the modified proteins into its core, where they are degraded [21, 22]. Ubiquitination, as a part of post-translational modification (PTMs), not only mediates protein degradation but also regulates protein activity and localization through monoubiquitination, multiubiquitination, or alternative polyubiquitination linkages, such as K63 and K27 [24, 25]. ALP utilizes several distinct mechanisms for transporting cargo to the lysosome for degradation [26]. One such mechanism is chaperone-mediated autophagy (CMA), which selectively targets proteins containing a specific pentapeptide motif (KFERQ-like sequence) into lysosomes via the translocation complex [27, 28]. Another mechanism is nonspecific macroautophagy, in which double-membrane vesicles, called autophagosomes, encapsulate damaged proteins and organelles and subsequently deliver them to the lysosome [27]. Additionally, there is microautophagy that uptakes of cytosolic material by invagination of the lysosomal or endosome membrane [29–31]. Moreover, other processes, such as endocytosis and micropinocytosis, also contribute to the delivery of extracellular and plasma membrane cargo to the lysosome [26]. Lysosomes, equipped with around 60 hydrolytic enzymes and maintaining an acidic pH of 4.5–5, break down macromolecules into basic units [32]. Generally, proteasome mediates the degradation of individual cellular proteins in a highly specific manner, whereas lysosome degrades cytoplasmic components, such as membrane proteins, protein aggregates, and defective or surplus organelles, primarily via autophagy and extracellular cargoes via endocytosis [33].

**Fig. 2:**
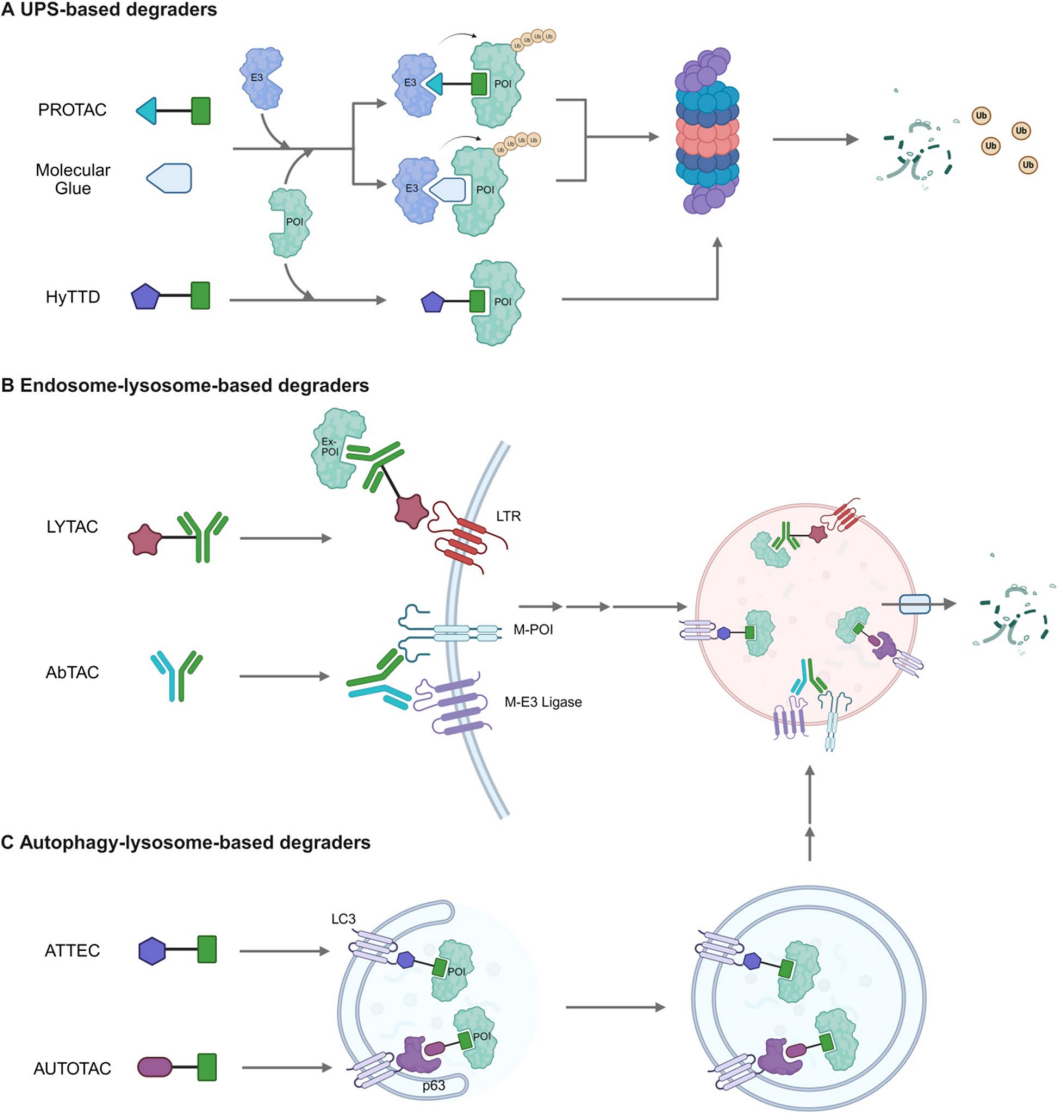
Targeted Protein Degradation Strategies. **A** The UPS-based degraders. PROTACs are bifunctional molecules that recruit the E3 ligase to POI, marking it for ubiquitination and subsequent proteasomal degradation. Molecular Glues can induce or stabilize interactions between proteins, leading to POI degradation. HyTTC employs hydrophobic tags attached to POI, mimicking misfolded proteins and triggering POI degradation by cellular quality control mechanisms [35]. **B** Endosome-lysosome-based degraders. LYTACs utilize ligand binding to LTRs, such as CI-MPR and ASGPR, mediating ex-POI degradation. Whereas CI-MPR is ubiquitously expressed in all human tissues, ASGPR is only expressed in the liver. AbTACs utilize a recombinant bispecific antibody to recruit the m-POI and the m-E3 ligase, RNF43 [35]. The POI is likely degraded via the lysosomes, but not by the proteasomes. However, the exact mechanisms remain to be established. **C** Autophagy-lysosome-based degraders. ATTECs simultaneously bind LC3 and the POI, while an AUTOTAC molecule binds p62 and the POI [35]. The binding induces the formation of autophagosomes, and subsequent fusion between autophagosomes and lysosomes leads to the POI degradation. LTRs, lysosome-targeting receptors; CI-MPR, cation-independent mannose 6-phosphate receptor; ASGPR, asialoglycoprotein receptor; Ex-POI, extracellular-POI; m-POI, membrane-bound protein; m-E3 ligase, membrane-bound E3 ligase; RNF43, ring finger protein 43; LC3, microtubule-associated protein 1 light-chain 3

Targeted protein degradation (TPD) is a sophisticated strategy that harnesses the cellular intrinsic protein degradation ability to selectively eliminate specific target proteins [34]. Initially, TPD involved recruiting a target protein to an E3 ubiquitin ligase. However, the field has since expanded to include various cellular degradation pathways [23, 35]. These pathways not only include UPS-based proteolysis-targeting chimeras (PROTACs) but also extend to ALP-based mechanisms, such as lysosome-targeting chimeras (LYTACs), antibody-based PROTACs (AbTACs), autophagosome-tethering compounds (ATTECs), and autophagy-targeting chimeras (AUTOTACs) [36, 37]. This expansion has significantly broadened the scope of targetable proteins to include extracellular proteins and cellular organelles (Fig. 2) [35, 38, 39]. Moreover, the development of specific molecules that induce degradation through mechanisms other than the direct recruitment of E3 ligases to the protein of interest (POI) has further diversified the TPD toolkit. Examples include molecular glues that modify protein conformation to enhance E3 ligase and POI interactions, as well as hydrophobic tag tethering degraders (HyTTDs) that mimic misfolded proteins [40–43].

PROTACs have been extensively studied and validated in both preclinical and clinical settings, with several candidates progressing to clinical trials [44]. However, the selection of its core component—E3 ubiquitin ligases—has long been restricted to cereblon (CRBN) and von Hippel-Lindau (VHL), which collectively account for less than 2% of the over 600 human E3 ligases [44–46]. This limitation presents multiple challenges for the clinical application of PROTACs, including drug resistance due to CRBN mutations in multiple myeloma and treatment failure resulting from VHL dysfunction in clear cell renal carcinoma [47, 48]. Consequently, researchers are intensively investigating the therapeutic potential of alternative E3 ubiquitin ligases.

Among the expanding repertoire of E3 ligases in PROTACs, Mouse double minute 2 (MDM2) has emerged as a therapeutically pivotal candidate due to its dual functionality [49–51]. MDM2 not only possesses intrinsic E3 ubiquitin ligase activity but also drives tumorigenesis and other diseases progression through both p53-dependent suppression and p53-independent activation pathways. This dual utility positions MDM2 at the forefront of PROTAC innovation in two distinct modalities, MDM2-harnessing PROTACs and MDM2-targeted PROTACs. The recent discovery of novel MDM2 recruitment strategies, spanning large molecules and natural product-inspired scaffolds, has significantly advanced both PROTAC categories. These ligands enable precise spatial orientation of ternary complexes, optimizing ubiquitination efficiency while minimizing off-target effects [52–54]. This review begins with an exploration of how the composition of PROTACs influences their design and therapeutic efficacy. We then provide a comprehensive overview of the structure and physiological functions of MDM2. Furthermore, we discuss the development of PROTACs that harness or target MDM2, highlighting their design features, functional properties, and potential applications in therapy.

## PROTACs

Since the concept of TPD was proposed in 1999, significant advancements have been made in the development of PROTAC molecules [33]. The first peptide-based PROTAC was synthesized in 2001, followed by the synthesis of the first small molecular PROTAC in 2008 [34, 55]. In recent years, there has been exponential growth in the development of PROTACs, with numerous candidates advancing to clinical trials and showing promising results [34, 55, 56]. A PROTAC molecule consists of a warhead that targets POI, an E3 ligase-recruiting ligand, and a flexible linker that connects the two. By forming a ternary complex of the target protein, PROTAC, and E3 ligase, PROTACs enable efficient target protein ubiquitination and subsequent degradation via the UPS. This mechanism allows for the selective elimination of previously undruggable proteins, marking a major therapeutic advancement [57–60].

### Selection and preference of POI and its ligand

According to PROTAC DB 3.0, the number of PROTACs has now reached 6,111, targeting 442 distinct POI targets through 20 different E3 ligases [61]. Proteins like the androgen receptor (AR), epidermal growth factor receptor (EGFR) mutants, cyclin-dependent kinases (CDKs), and Poly (ADP-Ribose) Polymerase 1 (PARP1) have been the focus of over 100 unique PROTAC designs [61]. These proteins, belonging to oncogenes, transcription factors, sex hormone receptors, or metabolic enzymes, have been extensively studied and well-represented in therapeutic development, with many inhibitors already established as clinical or preclinical therapeutics [61–65].

The selection criteria for POI in PROTAC design primarily depend on two interrelated factors: the target's functional centrality in disease pathogenesis and the druggability of existing pharmacological modulators [66]. This dual selection paradigm is particularly well-exemplified by protein kinases, which constitute over 50% of current PROTAC targets [61]. Protein kinases orchestrate critical cellular processes through phosphorylation-mediated regulation of signaling cascades, cell cycle dynamics, and metabolic reprogramming[67]. More critically, kinase dysregulation exhibits direct causal relationships with oncogenesis, and over 400 types of typical and atypical oral protein kinase inhibitors are currently undergoing clinical trials worldwide [68]. The structural conservation of ATP-binding pockets across the kinome has enabled systematic development of small-molecule inhibitors, particularly tyrosine kinase inhibitors (TKIs) targeting receptor tyrosine kinases (RTKs) [69, 70]. However, conventional ATP-competitive inhibitors frequently encounter clinical resistance through gatekeeper mutations or pathway reactivation, such as EGFR T790M/C797S in non-small cell lung cancer [71]. This therapeutic challenge creates a unique opportunity for PROTAC technology through two synergistic mechanisms. The catalytic degradation mode circumvents occupancy-driven resistance by eliminating both enzymatic and scaffolding functions of kinases. Meanwhile, the established chemical library of kinase inhibitors provides immediate starting points for PROTAC development, enabling strategic repurposing of suboptimal inhibitors that failed due to resistance mechanisms rather than target relevance [57].

Different with traditional small molecular inhibitors, the therapeutic efficiency of PROTACs is not solely dictated by the binding affinity of the POI-targeting warhead but is critically governed by optimal formation of the POI-PROTAC-E3 ligase ternary complex and complete exposure of ubiquitination-competent lysine residues on the POI surface [72–75]. Wurz et al. characterized the formation and degradation of ternary complexes involving SWI/SNF-related BAF chromatin remodeling complex subunit ATPase 2 (SMARCA2)-targeted and bromodomain-containing 4 (BRD4)-targeted PROTACs. They demonstrated that the binding affinity of the ternary complex (K_LPT_) and cooperativity (α) correlate well with the potency of degradation and the initial rates of degradation [76]. Specifically, cooperativity is defined as the ratio of the binding affinity of the binary to that of the ternary complex. Positive cooperativity (α > 1) indicates that the interface of the protein–protein interaction favors the formation of the ternary complex, which leads to more efficient degradation of the target protein. Therefore, even if the affinity of the POI warhead is low, it is still possible to design effective degraders and optimize pharmacokinetic (PK) properties after identifying cooperative molecules [77–80].

Conventional optimization of PROTAC often focuses on linker modification [61, 76, 81]. Although linkers can incorporate functional groups susceptible to oxidative metabolism via CYP450 enzymes, the inherent physicochemical properties of PROTACs, characterized by high molecular weight (> 700 Da), multiple hydrogen bond donors (HBDs), and low passive cell permeability, fundamentally alter their dominant elimination pathways [82]. Their placement within the"beyond Rule of 5"(bRo5) chemical space situates them near or above the glomerular filtration size threshold [75, 83]. Furthermore, their pronounced polarity significantly limits diffusion into cells, including hepatocytes, thereby attenuating the liver's role in oxidative metabolism as a primary clearance mechanism[75]. Consequently, structural modifications to warheads or linkers frequently exhibit minimal impact on systemic clearance, resulting in a shallow PK structure–activity relationship (SAR) [84, 85]. This observed flatness in PK SAR, however, does not diminish the importance of linker or warhead design. Instead, it necessitates a strategic shift in priorities: optimizing linkers through rigidification and hydrogen bond masking primarily aims to reduce polar surface area and enhance cellular permeability, rather than solely mitigating metabolic instability [82]. Once cellular penetration is enhanced, linker stability emerges as a primary driver of metabolic fate and PK properties. This transition explains why advanced PROTACs exhibit steeper SAR around linker modifications [86]. The permeability-first approach underpins the success of bRo5 PROTACs like ARV-471 and ARV-110, which achieve measurable oral bioavailability [87, 88]. For these optimized degraders, both oxidative and non-oxidative metabolism of the linker becomes the critical determinant of exposure.

The metabolic vulnerability of POI-targeting warheads may also dictate the overall stability of PROTAC molecules, a critical yet frequently underestimated design consideration. This intrinsic limitation is exemplified in AR-targeted PROTAC development, where the inherent instability of AR ligands imposes an upper boundary on PROTAC half-life regardless of linker/E3 ligase modifications [82]. Gabriele et al. systematically demonstrated this phenomenon using cryopreserved human hepatocyte models, the gold standard for hepatic metabolism prediction [82]. They found that the PROTACs series containing AR ligands have a half-life of under 100 min, which is consistent with the AR warhead’s short half-life of 18.3 min. Notably, despite alterations to the stable linkers and E3 ligase ligands, there has been no significant improvement in the metabolic stability of these PROTACs [82].

### Features and limitations of E3 ligases: VHL and CRBN

In the development of PROTACs, only a few E3 ligases have been widely used, namely VHL and CRBN, with others like MDM2 and cell inhibitor of apoptosis proteins (cIAP) being less accessible [46, 89–92]. The first PROTAC molecule utilized the phosphopeptide IkBα to recruit the Skp1-Cullin-F-box (SCF) ubiquitin ligase complex, facilitating the degradation of methionine aminopeptidase-2 (MetAP-2) [34]. However, the large size of this PROTAC limited its permeability into the cytoplasm, and the susceptibility of phosphopeptides to intracellular phosphatases further reduced degradation efficacy [92]. To overcome these challenges, Crews et al. utilized a peptide chain (ALAPYIP) derived from hypoxia-inducible-factor-1 α (HIF-1α) as a VHL ligand and added a Poly-D-arginine tag to the C-terminus of the peptide to enhance cell permeability in 2004 [93]. Between 2001 and 2008, peptide-based PROTACs generally succeeded in improving internalization into the cytosol, but the effective concentrations remained in the micromolar range. Additionally, peptides often exhibit poor water solubility and bioavailability, which limits their clinical applicability [55].

In 2008, Crews et al. developed the first small-molecule PROTAC [55]. This molecule utilized Nutlin-3a to recruit MDM2 and employed a polyethylene glycol (PEG)-based linker to combine with hydroxyflutamide, a non-steroidal androgen receptor ligand. However, its effective concentration remained in the micromolar range. Two years later, the first specific and non-genetic IAP-dependent Protein Eraser (SNIPPER) was developed, which recruited cIAP1 through methyl bestatin to degrade cellular retinoic acid binding proteins I and II (CRABP-I/II) [94]. The application of VHL small molecule inhibitors began in 2012, while the development of CRBN-based PROTACs started in 2015 [95, 96].

The core mechanism of PROTAC technology relies on E3 ligase-mediated ubiquitination and degradation of target proteins. However, over 98% of PROTACs in preclinical and clinical stages still exclusively utilize VHL or CRBN E3 ligases [97, 98]. While this narrow focus has accelerated early development, it severely limits the full potential of the technology. For instance, genetic mutations in *CRBN* have rendered lenalidomide-derived PROTACs ineffective in myeloma, highlighting the risks of single E3 dependency [47]. Since many experiments and clinical trials have demonstrated the adaptability of VHL-based and CRBN-based PROTACs, it would be beneficial to investigate the standard features of VHL and CRBN for further E3 ligase screening [23].

VHL has a well-documented ligand, the natural substrate peptide ALAPIYP (derived from HIF-1α peptide), whose high affinity makes it an attractive choice for PROTAC design [93]. The discovery of small molecule VHL ligands like VH032, VH101, and VH298 has further facilitated its widespread application in PROTACs [99]. Identifying IMiDs (such as thalidomide, pomalidomide, and lenalidomide) as ligands for CRBN was a significant breakthrough [96]. These ligands have high affinity and selectivity for CRBN, enabling the development of potent CRBN-based PROTACs. However, many other E3 ligases lack well-characterized and high-affinity ligands, limiting their application in PROTAC and TPD technologies [89]. Finding suitable ligands remains a critical step in PROTAC design, and the limited availability of ligands for other E3 ligases poses a significant challenge for their use in PROTAC development.

VHL and CRBN are ubiquitously expressed in various tissues throughout the body [97–99]. This widespread expression ensures that VHL-based and CRBN-based PROTACs can function effectively in different cellular environments, making them suitable for targeting proteins in multiple diseases [81, 89, 100]. Meanwhile, the subcellular locations of VHL and CRBN are mainly in the cytosol, and VHL can also shuttle to the nucleus, allowing them to be recruited for POI degradation by many PROTACs [101]. Sapkota et al. purposed that distinct locations of Halo or FKBP12^F36V^-tagged proteins (the degradation tag recognized by VHL-based or CRBN-based PROTACs) presented various degradation levels using the same respective chimeras, demonstrating that the subcellular context of the POI can influence the efficacy of PROTACs and proteins localized to the inner lumen of the Golgi appear to be resistant to be degraded by many PROTACs^104^. Therefore, when designing PROTACs, it is crucial to consider the subcellular distribution of both the E3 ligase and the POI. On the other hand, VHL and CRBN have low tissue specificity that impairs PROTACs from accumulating in the lesion, like solid cancer, and increases risks for side effects [102].

VHL and CRBN are among the most studied E3 ligases, especially regarding their partners and ubiquitination mechanisms [23]. This helps in the rational design of PROTACs, optimizing their linker and warhead components to enhance the stability and efficiency of the ternary complex formation. It has been proposed that physiological and natural partners or substrates of E3 ligases can either synergistically or antagonistically influence the degradation efficiency of PROTACs [102]. For example, pomalidomide-based PROTACs targeting Bruton’s tyrosine kinase (BTK) were found to degrade IMiD neo-substrates IKZF1, IKZF3, ZNF 827, and ZFP91, making unexpected side effects [103]. Although over 600 E3 ligases exist in human cells, many are involved in complex regulatory pathways. Various cellular signals and interactions may tightly regulate their functions, which adds to the complexity of harnessing them for PROTAC-mediated protein degradation [89, 104]. For example, Nedd4 family members maintain low enzymatic activity via phase separation in resting states to prevent non-specific substrate ubiquitination. This ensures they’re activated only under specific conditions [105]. However, the artificial interference of these E3 ligases needs to overcome the complexity of phase separation regulation, which increases the difficulty of developing PROTACs.

## Physiological and pathological functions of MDM2

The widespread use of E3 ligases such as VHL and CRBN in PROTAC technology has raised concerns about drug resistance [98]. Research has shown that after prolonged treatment, tumor cell lines can develop resistance to VHL-based and CRBN-based PROTACs. This resistance is mainly due to genomic alterations or a significant decrease in the expression of the *CRBN* gene or the *CUL2* gene, rather than point mutations in the residues that interact with their ligands or the POI [47, 98, 106, 107]. Meanwhile, the issue of tissue specificity has prompted research into alternative E3 ligases for PROTAC design [89]. MDM2, the E3 ligase used in the first small PROTAC molecule, has drawn renewed attention and investment due to its specific features and functions [50, 108].

### Structure and ubiquitination process of MDM2

Among mammalian cells, the p53 protein acts as the cellular envoy for the organism's well-being. When cells detect metabolic or genetic stress, p53 levels rise to halt the cell cycle and coordinate damage repair [109, 110]. MDM2 plays a key role in regulating p53 by binding to it and inhibiting its function. Specifically, MDM2 actively recruits enzymes that block p53-mediated transcription via methylation of histones. Meanwhile, MDM2 prevents p53 binding to p300/CBP, which are essential for transcription activation via histone acetylation [111]. Subsequently, MDM2 serves as an E3 ligase for p53 and facilitates its export from the nucleus to the cytoplasm and its degradation through the cytoplasmic proteasome. These continuous and effective actions of MDM2 control the lifespan of p53 short enough to ensure physiological activities in normal, unstressed cells [49, 112].

**Fig. 3:**
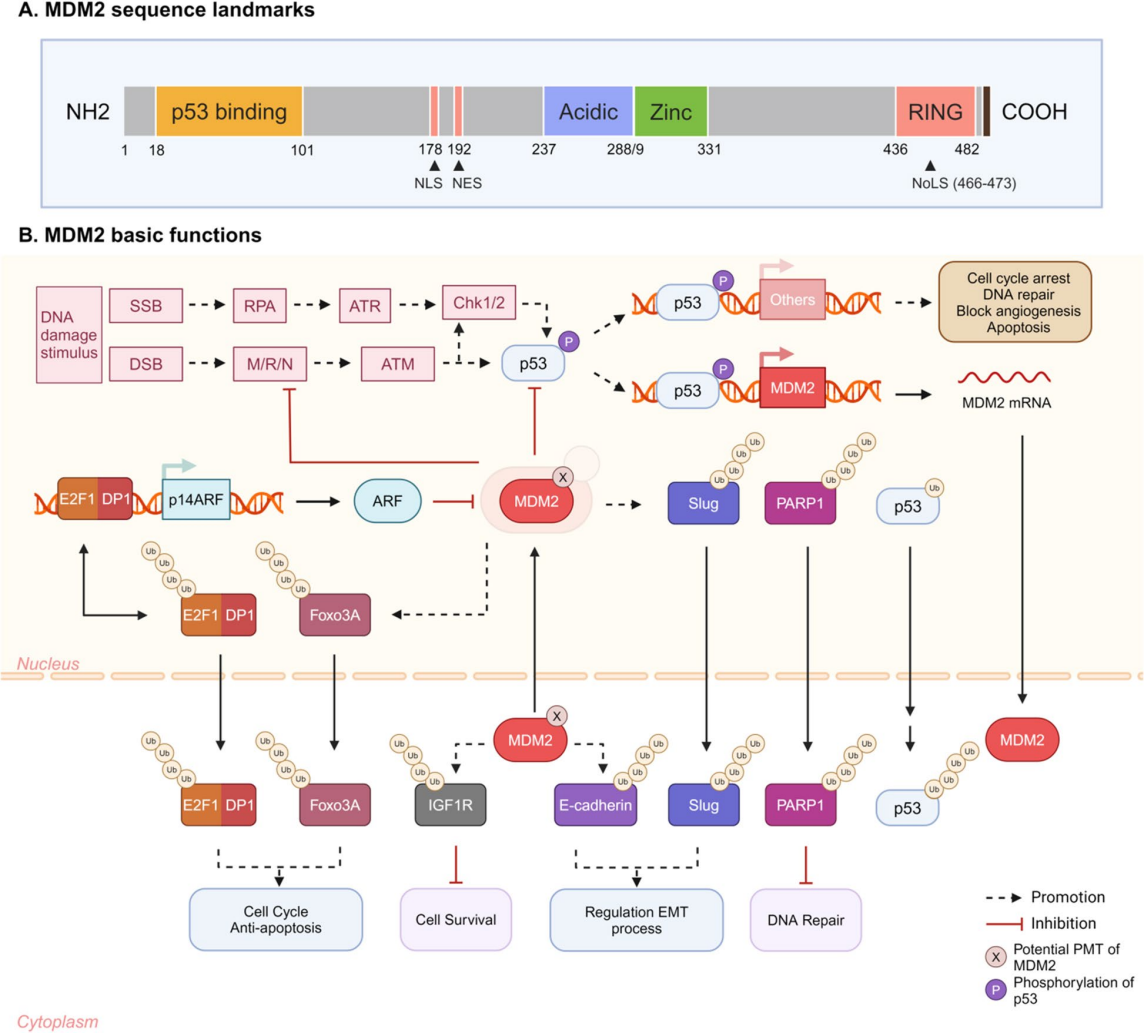
MDM2 Sequence and Its Function: p53 and Beyond. **A** MDM2 sequence landmark. *MDM2* is located on chromosome 12q15 and spans about 37 kb of genomic DNA. MDM2 is composed of 491 amino acids and contains three major functional regions: N-terminal p53-binding domain, central acidic domain, and C-terminal RING Finger domain. **B** MDM2 basic functions. With specific PTM, including multisites phosphorylation and acetylation, MDM2 is activated to recognize and regulate the functions of corresponding signal effectors via either facilitating ubiquitination or occupying their functional domains. Therefore, maintaining the physiological functions of MDM2 ensures cellular homeostasis, balanced stress responses, and tumor suppression

In human cells, MDM2 is a regulatory protein composed of 491 amino acids, forming multiple functional domains (Fig. 3A) [109]. These domains include the N-terminal p53-binding domain (residues 18–101), which inhibits p53’s transcriptional activity. A nuclear localization signal (NLS) at residue 178 and a nuclear export signal (NES) at residue 192 are critical for MDM2’s transport between the nucleus and cytoplasm. The acidic domain (residues 237–288) binds the ADP-ribosylation factor (ARF) and regulates p53 ubiquitination. The zinc-finger motif (residues 289–330) enables interactions with ribosomal proteins. The C-terminal RING domain (residues 436–482) contains E3 ubiquitin ligase activity, essential for p53 ubiquitination and the p53-MDM2 interaction; it also contains a nucleolar localization signal (NoLS) sequence. The carboxyl terminus tail (residues 485–491) facilitates MDM2 homodimer formation and MDM2-MDMX heterodimerization through interaction with the RING motif of another MDM2 or MDMX. However, the MDMX RING domain lacks intrinsic E3 ligase activity [111].

p53 is the natural substrate of MDM2, and the interaction between p53 and MDM2 has been generally identified. The co-crystal structure (PDB ID: 1YCR) shows that MDM2’s binding cavity is tailored to fit the amphipathic α-helix formed by p53’s transactivation domain [113]. The hydrophobic pocket of MDM2 interacts with the hydrophobic face of the α-helix, particularly engaging a triad of p53 amino acids–-Phe [19], Trp [23], and Leu [26]. This structural fit enables strong binding and causes a conformational change in MDM2, stabilizing the interaction. This binding inhibits p53’s transcriptional activation and triggers its ubiquitination and degradation. Moreover, MDM2’s central acidic domain and part of its zinc-finger motif interact with p53’s specific DNA-binding site, inducing a conformational change in p53 that exposes lysine residues for ubiquitination [114]. Besides, this weak binding participates in the regulation of ubiquitination signaling, as other proteins compete with p53 binding to the central region of MDM2 for prevention of p53 ubiquitination and degradation, such as p14ARF, and retinoblastoma (Rb) [115]. Additionally, a weak interaction between MDM2’s N-terminal and p53’s C-terminal promotes MDM2’s open conformation, enhancing its binding to p53’s transactivation domain [116]. The dynamic interactions between p53’s N-terminal or C-terminal domains and MDM2’s N-terminal domain influence modifications to p53’s C-terminal domain, particularly its lysine residues.

The key lysine for p53 ubiquitination is located at its extreme C-terminal domain, and both mono- and poly-ubiquitination contribute to its proteasome-mediated degradation [116]. For MDM2 to functionally interact with E2 enzymes like UbcH5c, it needs to form a RING dimer through MDM2 homodimerization or MDM2-MDMX heterodimerization [117]. However, research has proposed that the RING dimer is not sufficient for efficient p53 ubiquitination. And experiments have found that the fusion of the central acidic domain of MDM2 to the MDMX chimeric protein renders the protein fully capable of ubiquitinating p53 [114]. Additionally, a 30-residue region within MDM2’s central acidic domain interacts intramolecularly with the RING domain, stabilizing its conformation and enhancing E2 recruitment and ubiquitin transfer [118]. Monoubiquitinated p53 would expose the C-terminal NES to the surface and induce MDM2 dissociation [111]. Particularly, MDM2 contributes to the connection between p53 and small ubiquitin-like modifier (SUMO) E3 ligase PIASy, relying on K386 of p53, thereby facilitating p53 sumoylation, interaction with chromosomal region maintenance 1 (CRM1), and subsequently nuclear export [119, 120]. However, MDM2 seems to promote p53 nuclear export independently of sumoylation, as MDM2 could still enhance the nuclear export of the p53 K386R mutant, which lacks the SUMO modification site [120]. Therefore, this suggests two independent pathways for p53 nuclear export, one is sumoylation-induced p53 tetramer transportation, and another is MDM2-mediated monoubiquitination exporting signaling. Meanwhile, MDM2 also associates with 19S proteasome subunits, enhancing proteasome recognition of p53 post-ubiquitination [121]. It’s important to note that p53 remains as a tetramer when it moves from the nucleus to the cytoplasm. However, the 26S proteasome more readily recognizes and initiates degradation of p53 monomers that have been tagged with polyubiquitin chains [120]. The mechanism of p53 tetramer disassembly into monomers is still unclear and requires further investigation.

### Physiological functions of MDM2: independent of p53

MDM2 is a multifunctional protein that participates in various physiological processes independently of p53 (Fig. 3B). Beyond its role as an E3 ligase for p53, MDM2 can ubiquitinate other proteins. In the MAPK signaling cascade, MDM2 phosphorylates Foxo3A, a transcription factor involved in cell-cycle regulation, marking it for degradation [122]. MDM2 also interacts with the E2F1/DP1 complex, inhibiting its transcriptional activity and promoting its degradation, which prevents apoptosis and supports the G1-to-S phase transition of the cell cycle [123]. ARF, a downstream effector of the E2F1/DP1 complex, binds to MDM2 in the nucleolus, sequestering MDM2 and preventing it from interacting with other proteins [124–126]. Additionally, MDM2 ubiquitinates Slug and E-cadherin, which are involved in the epithelial-to-mesenchymal transition (EMT). Slug promotes EMT, and E-cadherin downregulation is a key consequence of EMT. This indicates that MDM2-mediated ubiquitination precisely regulates the progression of EMT by targeting both its initiators and downstream effectors [122, 127–129]. Studies have also shown that insulin-like growth factor 1 receptor (IGF-1R) competes with p53 binding to MDM2, making their relative abundance intricately linked. The downstream effector of IGF-1R, AKT, phosphorylates MDM2 to stabilize its ability to degrade p53. Thus, IGF-1R loss may cause p53 accumulation, leading to cell cycle arrest or apoptosis [125, 130, 131].

Considering that p53 transcriptionally activates MDM2 expression, a negative feedback loop is established, as MDM2 antagonizes p53 activity by promoting its degradation, which implies that MDM2 may be directly involved in the regulation of apoptosis and DNA damage repair [111, 132, 133]. When cells are exposed to ionizing radiation, introduced DNA double-strand breaks (DSBs) are detected by DNA damage sensors such as the MRE11-RAD50-NBS1 (MRN) complex and Ataxia Telangiectasia Mutated (ATM) kinase. These sensors initiate a signaling cascade that leads to the phosphorylation and stabilization of p53. However, under γ-irradiation, MDM2 is found to co-localize with MRN complex into DSB sites, and moreover, MDM2 only interacts with NBS1 through residues 198–314, without p53 or ARF participation, delaying DNA damage repair [134]. On the other hand, Matthias Dobbelstein’s group demonstrated that MDM2 directly binds and ubiquitinates PARP1, which is stimulated by DNA damage and helps recruit DNA repair proteins through the formation of poly(ADP-ribose) chains [135]. The accumulated evidence collectively indicates that MDM2 exerts regulatory functions in DNA damage repair without p53 participation.

### Roles of MDM2 in disease development and related targeting drugs

MDM2 abnormalities, including overexpression and mutations, manifest in both p53-dependent and p53-independent manners, extending beyond its well-known roles in physiological conditions. Its involvement in cancers, neurological disorders, cardiovascular diseases, and autoimmune diseases underscores its importance in maintaining cellular homeostasis and tissue integrity [136–139].

Since p53 is hard to target directly and MDM2 is involved in many diseases, MDM2 is a promising therapeutic target. Several inhibitors have been developed and entered clinical trials (concluded in Table 1) [151, 152]. Initial strategies focused on blocking the p53-MDM2 interaction based on structural insights. Many research groups developed peptide-based or small-molecule inhibitors to mimic p53's natural binding structure. The α-helix cyclic peptide ATSP-7041, which can inhibit both MDM2 and MDMX, was developed to overcome the conformational differences between short peptides and whole proteins. Currently, the optimized version of ATSP-7041, ALRN-6924, is in Phase I/IIa clinical trials for the treatment of solid tumors and lymphomas with wild-type TP53 (NCT02264613) [140]. When it comes to small-molecule inhibitors, the α-helix mimetics focused on key residues Phe [19], Trp [23], and Leu [26]. After high-throughput screening, cis-imidazoline analogs, known as Nutlins, were identified and found to effectively stabilize wild-type p53. The most biologically active Nutlin analog, Nutlin-3a, was subsequently optimized to yield the 2,4,5-triaryl imidazoline analog RG7112, sponsored by Roche’s ongoing phase I [142]. Of interest, researchers have proved that Nutlin-3 amplifies IGF-1R-Mdm2 association, affects IGF-1 signaling dynamics, and restrains cancer cell survival[130]. However, RG7112 exhibited significant limitations in phase I trials. Achieving efficacy required high doses that induced gastrointestinal toxicity, and therapeutic responses were inconsistent even at the maximum tolerated dose [153]. Another inhibitor, RG7388 (idasanutlin), with higher MDM2 affinity, was also developed but discontinued in Phase 3 trials for acute myeloid leukemia after failing to meet its survival goal when combined with cytarabine [143, 154].

**Table 1:** Different Inhibitors of MDM2

Structure	Inhibition effects	Mechanism of inhibition	Model & indication	Phase & NCT number	References
ALRN-6924	*TP53*-WT cell lines: IC_50_ < 1 μM; Carry mutant or null *TP53* cell lines: IC_50_ = 10–30 μM	Inhibition of p53-MDM2/X interaction via mimicking the N-terminal α-helical domain of p53	Prevention of Chemotherapy induced Myelosuppression	Phase 1 & NCT05622058	Pairawan et al. 2021 [140]
			Solid Tumor, Lymphoma, Lymphoma	Phase 1 & NCT03654716	
			Resistant (refractory) pediatric solid tumor, brain tumor	Phase 1 & NCT03654716	
Nutlin-3a	HOC-7, OVCA429, A2780: IC_50_ = 4–6 μM; SKOV3: IC_50_ = 38 μM; TOV21G: IC_50_ = 14 μM; OVAS: IC_50_ = 25 μM	Inhibition of p53-MDM2 interaction	MHM osteosarcoma, prostate cancer cell lines (LnCaP and 22Rv1)	/	Tovar et al. 2006 [141]
RG7112	SJSA1: IC_50_ = 0.3 μM RKO: IC_50_ = 0.4 μM HCT116: IC_50_ = 0.5 μM MDA-MB-435: IC_50_ = 9.9 μM	Inhibition of p53-MDM2 interaction	Sarcoma	Phase 1 & NCT01605526	Vu et al. 2013 [142]
			Neoplasms	Phase 1 & NCT00559533	
			Hematologic Neoplasms	Phase 1 & NCT00623870	
RG7388 (idasanutlin)	SJSA1: IC_50_ = 0.01 μM HCT116: IC_50_ = 0.01 μM	Inhibition of p53-MDM2 interaction	Solid Tumors	Phase 1 & NCT03362723	Ding et al. 2013 [143]
			Acute Myeloid Leukemia, Acute Lymphoblastic Leukemia, Neuroblastoma, Solid Tumors	Phase 2 & NCT04029688	
CP1-7C	MCF-7: IC_50_ = 2.5–5 µM DLD-1: IC_50_ = 2.5–5 µM SKOV-3: IC_50_ = ~ 10 µM HCT-116 (p53 WT): IC_50_ = ~ 5 µM HCT-116 (p53 Null): IC_50_ = ~ 10 µM	Inhibition of MDM2 E3 ligase activity via blocking the RING domain	Colorectal cancer, non-small cell lung cancer, Ovarian cancer, breast cancer	/	Singh et al. 2016 [144]
Serdemetan	OCI-AML-3: IC_50_ = ~ 0.2 µM MOLM-13: IC_50_ = ~ 0.3 µM NALM-6: IC_50_ = ~ 0.4 µM	Inhibition of MDM2 E3 ligase activity via preventing MDM2-proteasome interaction	Acute myeloid leukemia	Phase 1 & NCT00676910	Chargari et al. 2011 [145]
MEL23	293 T (Mdm2(wt)-luciferase): EC_50_ = 7.5 μM	Inhibition of MDM2-MDMX heterodimer E3 ligase activity without affecting MDM2 homodimer function	Effective in U2OS, MCF-7, H1299, RKO, RKO-E6, and HCT116 human cancer cell lines	/	Herman et al. 2011 [146]
MEL24	293 T (Mdm2(wt)-luciferase): EC_50_ = 9.2 μM		Effective in U2OS, MCF-7, H1299, RKO, RKO-E6, and HCT116 human cancer cell lines	/	Herman et al. 2011 [146]
MMRi62 (MMRi compounds)	Leukemic (NALM6, MV4-11, HL60, HL60VR, Primary AML): IC_50_ = 0.1–12 µM; Pancreatic cancer (Panc1, BxPc3, SW1990, panc10.05, HPAFII, AsPC-1, CaPan2): IC_50_ = 0.6–10.3 µM;	Inhibition of MDM2-MDMX heterdimer E3 ligase activity via blocking the RING domain of MDMX	Leukemic, pancreatic cancer	/	Li et al. 2022 [147]
SP141	Prostate cancer (HPAC, Panc-1, AsPC-1, Mia-Paca-2): IC_50_ = 0.4–0.5 μM; Breast cancer (MCF-7, MCF-7 p53-/-, MDA-MB-468, MDA-MB-231, MDA-MB-435): IC_50_ = 0.4–0.9 μM	Promotion of MDM2 autoubiquitination and degradation	Prostate cancer; breast cancer	/	Wang et al. 2014 [148]
MA242	Panc-1, Mia-Paca-2: IC_50_ = ~ 0.1 μM; AsPC-1: IC_50_ = ~ 0.2 μM; BxPC-3: IC_50_ = ~ 0.3 μM; HPAC: IC_50_ = 0.40 μM; HPDE: IC_50_ = ~ 5.8 μM	Promotion of MDM2 autoubiquitination and degradation via binding to C-terminal RING domain of MDM2	Pancreatic cancer	/	Wang et al. 2018 [149]
AQ-101	SUP-B13, EU-1, EU-3, EU-6, and EU-8: IC_50_ = 0.3–2.4 µM	Promotion of MDM2 autoubiquitination and degradation	Cell lines derived from children with ALL (cells contained different p53 status: WT, mutant, and null) and fresh leukemic samples	/	Gu et al. 2018 [150]

Inhibitors that block the p53-MDM2 interaction can maintain p53 activity but are only effective in tumors with wild-type p53, limiting their use in patients with p53 mutations [155–157]. Moreover, since MDM2 is involved in many p53-independent regulatory pathways, targeting only the p53-MDM2 interaction may not fully harness MDM2's potential. Alternative approaches that directly reduce MDM2 expression, enzyme activity, or protein levels are needed. Targeting MDM2 transcription, while aiming to elevate p53 levels, ultimately faces the challenge of reintroducing the complexities of p53 inhibition and the intricate regulation of epigenetics [158]. For instance, Adriamycin (doxorubicin) downregulates MDM2 but not through direct transcriptional inhibition [151]. Serdemetan (JNJ-26854165), an E3 ligase activity inhibitor, prevents MDM2-proteasome binding but has limited clinical use due to toxicity and off-target effects [145]. Another inhibitor, MEL23/24, specifically targets the MDM2-MDMX heterodimer's enzyme activity and works synergistically with DNA-damaging agents [146]. On the other hand, MMRi compounds and CPI-7C inhibit MDM2 E3 ligase activity by blocking to the RING domain, preventing both homodimer and heterodimer complex formation, and inducing the degradation of MDM2 and MDMX [144, 147]. CPI-7C also blocks MDM2's p53 binding domain, protecting p53 from degradation. Since MDM2 dimerization activates its ubiquitin transfer ability, targeting the autoubiquitination pathway has led to the development of additional inhibitors, such as SP141, MA242 and AQ-101 [148–150].

## MDM2-harnessing PROTACs

**Fig. 4:**
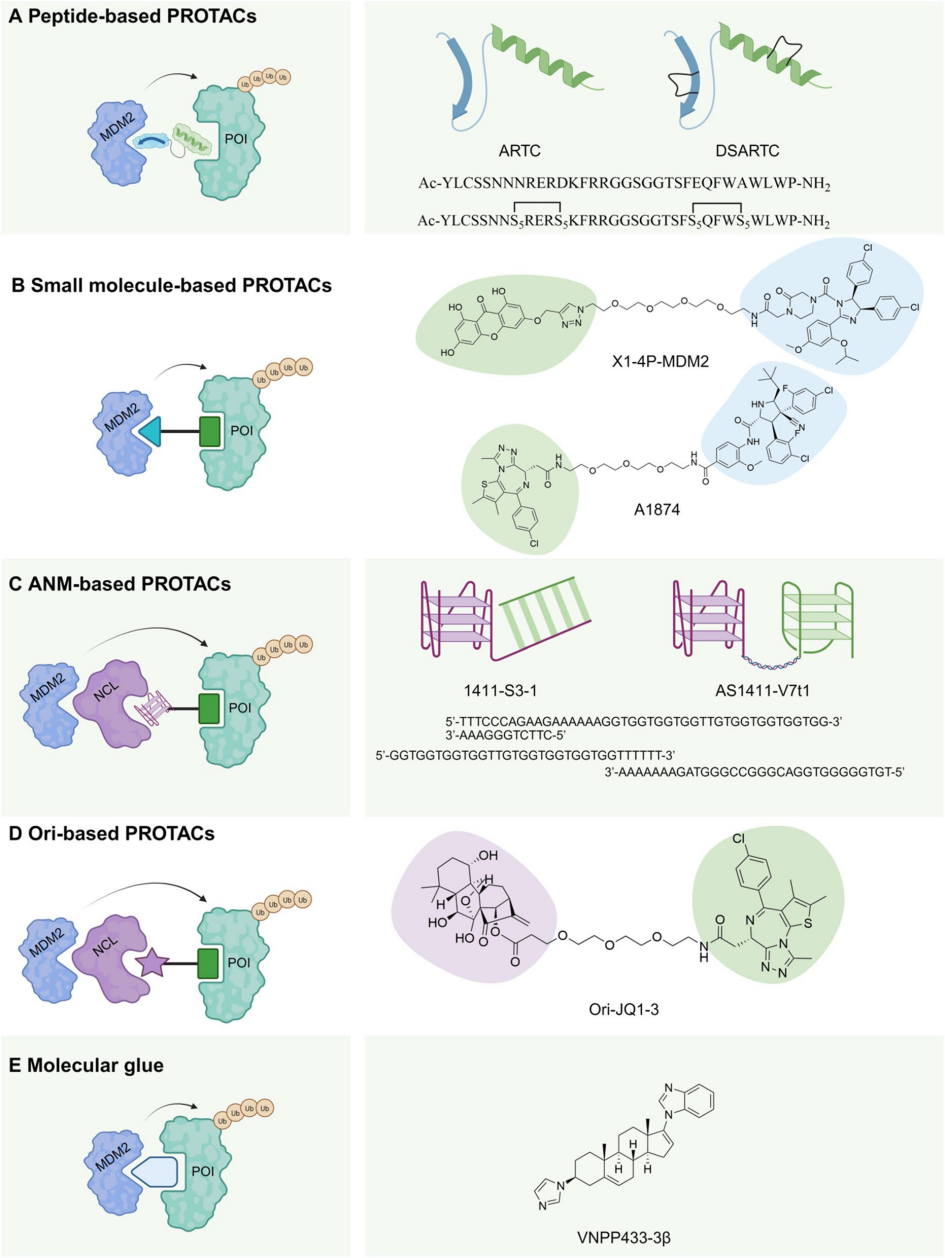
MDM2-harnessing PROTACs and molecular glue. Four distinct types of MDM2-harnessing PROTACs are classified by their mechanisms of recruiting MDM2. These recruitment strategies are as follows: **A** Peptide-based PROTACs. Representatives of this group include ARTC and optimized DSARTC designed to degrade AR and AR-V7. **B** Small molecule-based PROTACs. Instances include X1-4P-MDM2 and A1874 engineered to degrade DYRK1A and BRD4, respectively. **C** ANM-based PROTACs. They are exemplified by AS1411-S3-1 and AS1411-V7t1, degrading STAT3 and VEGF165, respectively. **D** Ori-based PROTACs. They are presented by Ori-JQ1-3, degrading BRD4. Another type of degraders utilizing UPS: **E** Molecular glues. VNPP433-3β is a prime example of them that specifically facilitates the interaction between MDM2 and AR/AR-V7

Given the availability of inhibitors with high binding affinity, MDM2 is a suitable candidate for the development of PROTACs [157, 159, 160]. Although the first pioneering small molecule PROTAC utilized MDM2 as an E3 ligase via Nutlin-3a in 2008, significant advancements in MDM2-based PROTACs have occurred since 2018, paralleling the broader growth in the PROTAC field [54, 55]. Correspondingly, various MDM2 inhibitors, including peptide-based and small-molecule types, have been incorporated into PROTAC designs and applied to multiple cancers and diseases (Fig. 4 and the details in Table 2).

**Table 2:** MDM2-harnessing and MDM2-targeted degraders

Name	POI (ligand)	E3 Ligase (ligand)	Model & indication	Efficiency	References
ARTC	AR and AR-V7	MDM2	Prostate cancer	LNCaP: DC_50_ = 175 nM C4-2: DC_50_ = 193 nM CWR22rv1: DC_50_ = 49 nM (AR)/79 nM (AR-V7)	Ma et al. 2022 [161]
DSARTC	AR and AR-V7	MDM2	Prostate cancer	C4-2: DC_50_ = 2.7 μM LNCaP: DC_50_ = 1.9 μM CWR22Rv1: DC_50_ = 2.5 μM (AR)/1.2 μM (AR-V7)	Ma et al. 2024 [162]
X1-4P-MDM2 DYRK1A degrader	DYRK1A (desmethylbellidifolin)	MDM2 (Nutlin-3)	T1DM and T2DM	INS-1: IC_50_ = 1.4 μM DC_50_, 48 h = 2.3 μM Dmax, 48 h = 83%	Yang et al. 2024 [163]
A1874	BRD4 (JQ1)	MDM2 (idasanutlin)	Colorectal cancer, lung cancer, osteosarcoma, leukemia, melanoma	HCT116: DC_50_ = 32 nM, IC50 = 86 nM SJSA1: IC_50_ = 47 nM A375: IC_50_ = 236 nM	Hines et al. 2019 [45]
Nutlin-3 based Homo-PROTAC	MDM2 (Nutlin-3)	MDM2 (Nutlin-3)	Non-small cell lung cancer, Liver cancer, colorectal cancer, breast cancer	A549: IC_50_ = ~ 1.0 μM HepG2: IC_50_ = ~ 3.3 μM HCT116: IC_50_ = ~ 1.9 μM MCF-7: IC_50_ = ~ 2.9 μM	He et al. 2020 [164]
VNPP433-3β	AR and AR-V7	MDM2	Prostate cancer	LNCaP: GI_50_ = 0.2 μM C4-2B: GI_50_ = 0.3 μM CWR22Rv1: GI_50_ = 0.3 μM	Retheesh et al. 2023 [165]
ursane-thalidomide-based PROTAC	MDM2 (Ursolic acid)	CRBN (Thalidomide)	Non-small cell lung cancer, hepatocellular carcinoma, lung cancer	A549: IC_50_ = ~ 0.2 μM Huh7: IC_50_ = ~ 0.4 μM HepG2: IC_50_ = ~ 0.4 μM	Zhiwen et al. 2021 [166]
GAA PROTAC: V10	MDM2 (ganoderic acid A)	VHL (VH032)	breast cancer, osteosarcoma, hepatocellular carcinoma	MDA-MB-231: DC_50_ = 80 μM	Li et al. 2024 [167]
MD-244	MDM2 (MI-1242)	CRBN (lenalidomide)	Leukemia cell	RS4;11(p53 wt): IC_50_ = 2–4 nM RS4;11(p53 mut): IC_50_ > 1000 nM	Li et al. 2019 [168]
MD-265	MDM2 (MI-1061)	CRBN (lenalidomide)	Leukemia cell, breast cancer	RS4;11: IC_50_ = 0.7 nM MV4;11: IC_50_ = 2 nM	Angelo et al. 2024 [169]
MG-277	GSPT1 (MI-2103)	CRBN (lenalidomide)	Leukemia cell, Breast cancer	RS4;11: IC_50_ = 3.5 nM, DC_50_ = 1.3 nM RS4;11/IRMI-2 cells: IC_50_ = 3.4 nM, DC_50_ = 2.5 nM	Yang et al. 2019 [170]
KT-253	MDM2 (MI-1061 analog)	CRBN (lenalidomide analog)	high-grade myeloid malignancies, solid tumors, lymphomas	HEK293T: DC_50_ = 0.4 nM, RS4;11: IC_50_ = 0.3 nM	Chutake et al. 2025 [171]
MD-4251	MDM2 (MI-1061)	CRBN (lenalidomide analog)	Leukemia cell	RS4;11: IC_50_ = 1 nM, AUC_0−24 h_ = 20,658 h·ng/mL, Oral bioavailability = 39% in mice	Acharyya et al. 2025 [86]
1411-S3-1	STAT3 (a decoy ODN)	MDM2 (AS1411-NCL)	Cervical carcinoma	Hela: DC_50_ = 136 nM	Li et al. 2024 [172]
HomoAS1411	MDM2 (AS1411-NCL)	MDM2 (AS1411-NCL)	Cervical carcinoma	Hela: DC_50_ = 71 nM	Wang et al. 2024 [173]
AS1411-V7t1	VEGF165 (V7T1)	MDM2 (AS1411-NCL)	Cervical carcinoma	HeLa: DC_50_ = 109 nM	Feng et al. 2025 [174]
Ori-JQ1-3	BRD4 (JQ1)	MDM2 (Oridonin)	Liver cancer	DC_50_ = 100–250 nM	Huang et al. 2025 [175]
Ori-Ori	MDM2 (Oridonin)	MDM2 (Oridonin)	Liver cancer	DC_50_ < 200 nM	Huang et al. 2025 [175]

### Conventional peptide-based PROTACs

Peptide-based ligands, despite having poor cell permeability and a short half-life that can limit their therapeutic effectiveness, offer advantages such as high binding affinity, specificity, and the ability to target proteins that are difficult to drug [176–178]. In 2022, Li et al. focused on targeting the DNA-binding domain (DBD) of AR. This domain is crucial for the transcriptional activity of both full-length AR and its splice variant AR-V7, which are key factors in castration-resistant prostate cancer (CRPC) [161]. The DBD is highly conserved across different AR mutants and variants but is distinct from other hormone receptors. It has a flat, extended protein–protein interaction surface that favors dimerization, making it difficult for small molecules to bind but accessible to peptide drugs. This study design used an AI system Rosetta to screen and identify high-affinity binding sequences for the AR DBD and MDM2, significantly speeding up the drug discovery process. The researchers evaluated the affinity of the final selected peptide chains for the two substrates by isothermal titration calorimetry with a dissociation constant (Kd) of 12.2 nM for MDM2 and 49.6 nM for AR-V7 [161]. To enhance the stability and cell penetration of their PROTAC, they conjugated peptides to Au nanoparticles, forming Au-AR pep-PROTAC, which has a half-life of 26.3 h. This complex effectively induced degradation of both AR and AR-V7 in prostate cancer cell lines, with IC50 values ranging from ~ 130 nM to ~ 250 nM.

Inspired by ALRN-6924 and the advantages of hydrocarbon-stapled peptides, Lei Li et al. later designed a double-stapled peptide named double-stapled AR targeting peptide PROTAC (DSARTC) in 2024 to recruit AR and MDM2 [162]. Hydrocarbon stapling is a technique used to enhance the stability and functionality of peptides by introducing covalent cross-links (staples) that rigidify the peptide backbone. This study introduced the first"first-in-class"heterogeneous-conformational double-stapled peptide, using hydrocarbon stapling to stabilize both β-sheet and α-helix structures within a peptide. The DSARTC molecule replaces the N-terminal Asn8-Asp12 and C-terminal Glu25-Ala29 in the original sequence with two S5-S5, paired S-2-(4'-pentenyl) alanine. As expected, the staple-modified peptide shows better thermal stability (Tm = 59.6℃) and improved resistance to serum and enzyme degradation without affecting ternary complex formation [162]. In a xenograft mouse model of prostate cancer, DSARTC exhibits prolonged tumor retention (over 48 h), suggesting improved target engagement and reduced off-tumor toxicity.

### Conventional small molecule-based PROTACs

The choice of E3 ligase in specific diseases is crucial for degradation efficacy and therapeutic outcomes. For example, Chen et al. designed degraders for DYRK1A by linking its inhibitor desmethylbellidifolin analog X1 with six different E3 ligase ligands, including CRBN, VHL, cIAP1, and MDM2 [163]. In recent years, β-cell deficiency and dysfunction have been identified as key factors affecting both insulin production and resistance, contributing to the development of type 1 diabetes mellitus (T1DM) and type 2 diabetes mellitus (T2DM) [179, 180]. DYRK1A functions as a critical regulator in islet beta cell proliferation by phosphorylating key components of the cell cycle checkpoint control system. This phosphorylation inhibits the activity of proteins like NFATc1 and p27, and can lead to the degradation of proteins such as Cyclin D1 and the insulin receptor substrate-2 (IRS-2) [181]. Inhibiting DYRK1A removes these constraints, activating regenerative pathways and offering potential for diabetes treatment. Among 28 designed PROTACs, Chen’s team found that X1–4P-MDM2 was the most effective, achieving ~ 83% degradation efficiency at 48 h and significantly promoting β-cell proliferation. Notably, while cIAP1-recruiting PROTACs showed higher inhibition efficiency, they lacked degradation potency even at 20 μM.

Incorporating MDM2 into PROTAC technology offers significant advantages, particularly regarding p53 binding. In 2019, Crews et al. compared two PROTACs, A1874 (recruiting MDM2) and A743 (recruiting VHL), to highlight the benefits of using MDM2 in PROTACs [45]. When MDM2 participates in the ternary complex for POI ubiquitination, it disrupts its interaction with p53, preventing p53 degradation and leading to p53 stabilization and accumulation in cells. A1874 demonstrated synergistic effects through both BRD4 degradation and p53 stabilization, especially effective in cell lines with wild-type p53. Although both A1874 and A743 achieved nanomolar potency in BRD4 degradation (DC50 values of ~ 32 nM and ~ 23 nM, respectively), A1874 showed greater efficacy in reducing cancer cell viability [45]. For instance, A1874 was measured to inhibit 97% of cell viability in the HCT116 cell line, compared to 69% for A743. They also compared A1874 with A1875, which has specific stereochemical changes in its idasanutlin component [45]. These changes reduced MDM2's binding affinity for A1875, making it less effective at protein degradation. Even at a concentration of 10 μM, A1875 showed no significant degradation of BRD4 in HCT116 cells.

Consistent with the utilization of the UPS in PROTACs, molecular glue degraders are monofunctional small molecules that bind to either the E3 ligase or the target protein, facilitating the formation of a neo-interface between the E3 ligase and the target protein without significant affinity for the other partner [23, 182, 183]. Researchers have demonstrated that the efficacy of degraders is fundamentally linked to ternary complex binding affinity and cooperativity, underscoring the importance of the interaction between the target protein and the E3 ligase in determining the overall degradation potency of the degraders [76]. Generally, the rational design of molecular glues needs high-throughput screening platforms focusing on targets and their effectors together. However, some of the molecular glues were identified by chance [184–187]. For example, VNPP433-3β, a next-generation galeterone analog, was found to bind the AR and promote its degradation in prostate cancer cell lines [165]. Although the potential mechanism by which VNPP433-3β facilitates AR degradation is not completely clear, a recent study has provided evidence that VNPP433-3β serves as a molecular glue that effectively degrades AR and AR-V7, thereby inhibiting oncogenic signaling pathways in prostate cancer cells [165].

### A new type of NCL-bridged PROTACs

In light of the challenges associated with cellular permeability, the recruitment of PROTAC molecules to the sites of action and their subsequent therapeutic efficacy often fall short of expectations [188, 189]. This limitation restricts the broader application of PROTAC technology in clinical settings. Our group has noticed the natural interaction between MDM2 and nucleolin (NCL), and this binding does not affect MDM2 autoubiquitination ability [190]. In alignment with MDM2, NCL is frequently overexpressed in various cancer types, and it exhibits a dynamic shuttling mechanism between the nucleus, cytoplasm, and cell surface, as well as serving as a membrane-anchored receptor in both tumor cells and endothelial cells of angiogenic blood vessels [191–193].

#### ANM-based bridged PROTACs

The AS1411 aptamer, a G-quadruplex (G4) oligonucleotide, was discovered by chance and has demonstrated high-affinity and specific binding to NCL [193]. This G4 aptamer can efficiently enter cells through the endocytosis pathway. By binding to NCL, it redirects NCL away from its normal cellular functions, resulting in antineoplastic and antiviral effects [191]. However, despite showing promise in phase I and II clinical trials, AS1411 failed in phase III trials due to insufficient response rates and suboptimal PK properties [194].

The functional efficacy of AS1411 is attributed to its central region, which permits modifications to the 5'and 3'termini without compromising its binding affinity for NCL [191]. Leveraging this unique property, extensive research has repurposed AS1411 as a component in the development of novel therapeutic agents through its conjugation with an array of small molecules, aptamers, and nanoparticles [195–197]. These conjugates have demonstrated enhanced efficacy in targeting specific lesions, including an improved ability to permeate the blood–brain barrier [191]. Furthermore, beyond serving as the POI warhead in NCL-targeted PROTACs, AS1411 has been conjugated with a BRD4-targeting PROTAC molecule as an additional moiety [198]. This dual-action strategy successfully augments therapeutic efficacy and reduces side effects. The integration of AS1411 into PROTAC design not only capitalizes on its role as a functional backbone but also suggests its potential utility in the advancement of PROTAC technology.

Following the estimation of the feasibility of the ternary complex AS1411-NCL-MDM2 formation within the cytoplasm, our group hypothesized that this aptamer could be utilized in PROTAC design to enhance cellular permeability and lesion targeting [172]. Unlike traditional PROTACs that form ternary complexes (E3 ligase-PROTAC-POI), AS1411-NCL-MDM2 (ANM)-based PROTACs mediate the formation of quaternary complexes to close E3 ligase and POI together through the molecular bridge NCL. Given the high internalization efficiency of AS1411, we initially conjugated it with large-molecular-weight, cell-impermeable ligands for the selected"undruggable"POIs: transcription factor decoy oligodeoxynucleotides (ODNs) targeting STAT3 and c-Myc; an RNA aptamer for p53-R175H; and a long non-coding RNA (lncRNA) SLNCR1-derived RNA ligand for AR-V7 [172]. These POI warheads are therapeutic candidates that can specifically bind and neutralize their intracellular molecular targets but are ineffective without transfection or microinjection. Measurements of degradation efficiency and antitumor capacity in vitro and in vivo revealed that these repurposed degraders function effectively through simple incubation in vitro and intravenous administration in vivo*,* even outperforming small-molecule PROTACs.

Considering the convenient synthesis of oligonucleotides, further modification of AS1411 with oligonucleotides is more straightforward than small molecules, potentially reducing the cost of preclinical trials. Our group then explored the impact of the sequential order of AS1411 and oligonucleotide V7t1, another G4 aptamer that binds to VEGF165 [174]. Specifically, V7t1 was conjugated to either the 3'end or the 5'end of AS1411, resulting in two constructs: AS1411-V7t1 and V7t1-AS1411, respectively. While both constructs exhibited comparable binding affinities to tumor and vascular endothelial cells, AS1411-V7t1 was demonstrated to have superior degradation potency. This was evidenced by a lower DC_50_ value and more pronounced pro-apoptotic and anti-angiogenic effects in vitro. Furthermore, AS1411-V7t1 presented enhanced stability during serum incubation, suggesting that the G4 structure of V7t1 may be compromised in V7t1-AS1411, whereas the structure of AS1411 remains intact in both PROTACs [174].

#### Ori-based bridged PROTACs

Given the significant efficacy of ANM-PROTACs, our group aimed to enhance their bioavailability and explore oral administration by replacing the AS1411 aptamer with alternative NCL ligands, specifically Oridonin (Ori) and iSN04 [175]. Ori is a natural diterpene compound from the traditional Chinese herb *Rabdosia rubescens* that reversibly binds to NCL. iSN04 is a recently identified ssDNA aptamer that recognizes NCL and is smaller than AS1411. Initial evaluations showed that iSN04 disrupts the crucial NCL-MDM2 interaction, so it was excluded. Ori, however, maintains this essential interaction, making it more suitable for PROTAC design. Consequently, we synthesized a series of Ori-based PROTACs targeting key oncogenic proteins, including BRD4, EGFR, and MDM2 itself, to demonstrate proof-of-concept [175]. This study revealed that Ori-based PROTACs significantly enhance cellular uptake through both NCL-dependent internalization and NCL-independent diffusion, attributed to their smaller size. This dual mechanism results in higher potency for degrading POIs. For example, Ori-JQ1-1, targeting BRD4, exhibited a DC_50_ value of 200 nM, which is notably lower than the 400 nM required for AS1411-JQ1 to achieve similar effects. Considering the diversity of Ori binding proteins, the chemical proteomic analysis explored that Bio-Ori-1 could capture identified substrates exportin 1 and 3-phosphoglycerate dehydrogenase, while neither of them is an E3 ligase to interfere with the establishment of the degradation system [175]. However, further optimization is needed for Ori-based PROTACs, particularly in linker length and composition, to improve efficacy and stability. Developing modified Ori analogs with better solubility and bioavailability could also enhance the therapeutic efficacy of these PROTACs.

## MDM2-targeted PROTACs

**Fig. 5:**
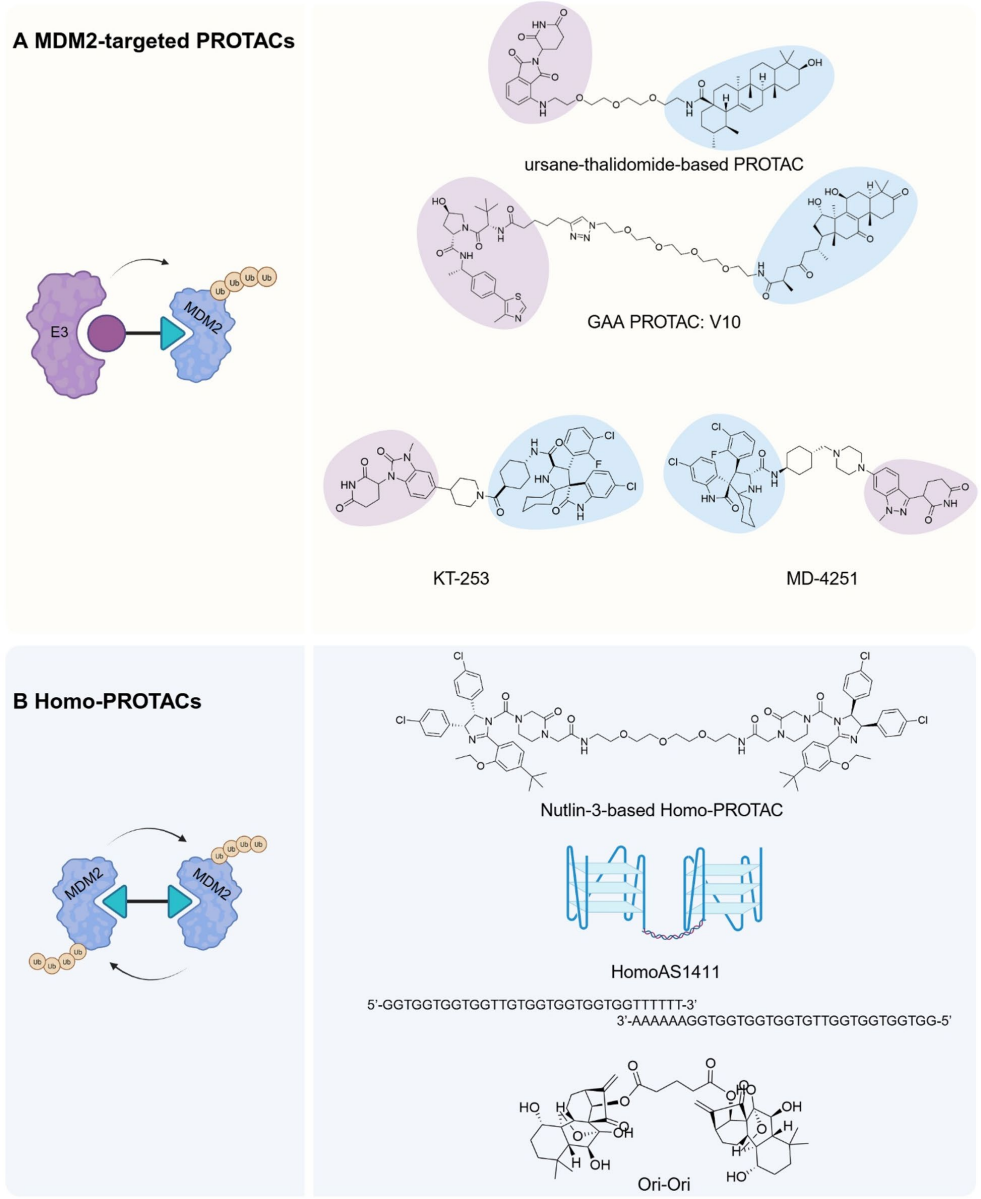
The MDM2-targeted PROTACs. Two types of MDM2-targeted PROTACs are classified by the selection of E3 ligases promoting MDM2 degradation. **A** MDM2-targeted PROTACs. Representatives of this group include ursane-thalidomide-based PROTAC, GAA PROTAC: V10, KT-253 and MD-4251, which recruit either CRBN or VHL as E3 ligase for MDM2 ubiquitination. **B** Homo-PROTACs. Instances include Nutlin-3-based Homo-PROTAC, HomoAS1411, and Ori-Ori, inducing MDM2 autoubiquitination and suicide in different recruiting mechanisms

MDM2 plays a crucial role in various diseases, making it a prime target for developing new PROTAC molecules. These molecules are designed to selectively degrade MDM2, modulating its activity and reducing its harmful effects [108, 199]. MDM2-targeted PROTACs come in different forms, including those based on CRBN and VHL E3 ligases, as well as homo-PROTACs that promote self-degradation of MDM2 (Fig. 5) [159]. Besides, these PROTACs also serve as powerful tools to compare enzyme activities by mediating the crosstalk between different E3 ligases, which might be helpful for PROTAC design and future optimization.

### Conventional CRBN- and VHL-based PROTACs

Natural products and their derivatives have emerged as promising candidates in the design of MDM2-targeted PROTACs, offering alternatives to peptide-based and small-molecule-based MDM2 ligands. In 2021, Wang et al. utilized ursolic acid (UA), a naturally occurring pentacyclic triterpenoid known for its antitumor properties and its MDM2-binding ability, to design a series of PROTAC molecules with CRBN ligand thalidomide [159, 166]. Among 6 PROTAC types, those with shorter polyoxyether linkers showed better efficacy, achieving an IC50 of 230 nM against the A549 cell line. Furthermore, Ganoderic acid A (GAA), the most abundant triterpenoid component in *Ganoderma lucidum*, has been recognized for its antitumor activity through its inhibition of activator protein 1 (AP-1) and nuclear transcription factor-κB (NF-κB) [167]. Based on GAA's MDM2-binding affinity, Tian et al. in 2024 developed PROTACs by linking GAA with thalidomide or the VHL ligand VH032 [159, 167]. After comparing 20 PROTACs, they found that GAA-VH032 with triazole-PEG linkers showed enhanced anticancer activity. Although these PROTACs showed low toxicity in zebrafish, their effective concentrations remained in the micromolar range *in vitro *[167].

Since the synthesis of PROTAC MD-224 by conjugating the MDM2 inhibitor MI-1242 with CRBN ligand lenalidomide in 2018, Wang et al. have utilized it as a lead compound for further modifications, developing new degraders [168]. The team optimized MD-224 into MD-265 by replacing the phenyl group of MI-1242 with a trans-cyclohexyl group and adding piperidine to the linker. MD-265 can effectively degrade MDM2 and strongly increase p53 and its downstream effectors even at a concentration of 1 nM [169]. However, upon replacing the benzoic acid fragment in MI-1061 with a methyl group to yield MI-2103, researchers observed that not only MI-2103 exhibit approximately five-fold reduced potency in binding to MDM2 compared to MI-1061, with IC_50_ values of ~ 9.5 nM and ~ 48 nM, respectively, but the related PROTAC MG-277 also showed diminished efficacy in degrading MDM2 [170]. Intriguingly, MG-277 has the ability to bind the translation termination factor G1 to S phase transition 1 (GSPT1) and mediate its interaction with CRBN, facilitating its degradation. Thus, MG-277 was identified as a molecular glue, with DC_50_ values of 1.3 nM in the RS4;11 cell line with 24 h treatment.

The first-in-class MDM2 degrader KT-253 (NCT05775406) is under clinical evaluation for high-grade myeloid malignancies, solid tumors, and lymphomas [200]. Preclinical data demonstrate exceptional potency: KT-253 achieves a DC_50_ of 0.4 nM in HEK293T cells after 4-h exposure and induces sustained tumor regression in RS4;11 acute lymphoblastic leukemia (ALL) and MV4;11 acute myeloid leukemia (AML) xenograft models at a single intravenous dose of 3 mg/kg [171]. Critically, unlike traditional MDM2 inhibitors, KT-253 shows no neutropenia or thrombocytopenia in advanced lymphoma/ALL patients, addressing a major toxicity limitation of occupancy-driven inhibitors [200]. However, the above MDM2 degraders exhibit critically low oral bioavailability, even < 2% in the mouse model, substantially limiting their therapeutic utility [86]. Wang et al. systematically optimized the linker and tethering position of lenalidomide-derived MD-6126, achieving 11.4% oral bioavailability in mice while maintaining sub-nanomolar degradation potency in RS4;11 cells. Building on this, iterative screening of CRBN ligands and linkers yielded MD-4251, the first orally bioavailable MDM2 degrader [86, 201]. In murine PK studies, a single 3 mg/kg oral dose achieved 39% bioavailability [86, 201]. Notably, a single 50 mg/kg oral dose induced complete tumor regression in leukemia xenografts, while 60 mg/kg elicited no platelet depletion in female mice [86]. These data position MD-4251 as a promising clinical candidate for p53-wild-type hematologic malignancies.

### Conventional homo-PROTACs

Homo-PROTACs exemplify the integration of MDM2's dual functionality by inducing its self-degradation [164]. Targeting MDM2's E3 ligase activity offers a therapeutic strategy beyond just blocking its interaction with p53, as MDM2 can degrade other proteins like Foxo3A and the E2F1/DP1 complex [122]. Moreover, MDM2's natural homodimerization activates its enzyme activity and facilitates autoubiquitination, reducing the risk of declining E3 activity in the ternary complex [111]. Sheng et al. constructed homo-PROTACs using two Nutlin-3-derived molecules to induce MDM2 self-degradation [164]. This design recruits two MDM2 molecules through identical backbones, minimizing off-target effects. The synthesis of these homo-PROTACs involves various linker systems, which significantly influence MDM2 binding affinity, degradation efficiency, and antitumor activity. The analyses of MDM2-p53 competitive binding activity revealed that extended alkyl linkers are detrimental to effective MDM2 binding, whereas the substitution of alkyl chains with PEG chains enhances binding activity [164]. Conversely, shortening the PEG chain substantially diminishes binding affinity. The addition of a flexible imino group at the end of the PEG chain in PROTACs increases their binding affinity. However, this particular PROTAC shows only moderate degradation efficiency, which might be due to reduced cell permeability. Sheng et al. have also isolated the enantiomers from synthesized PROTACs and identified one molecule that has superior antitumor effects both in vitro and *in vivo *[164].

### NCL-bridged homo-PROTACs

Besides direct recruitment pathways, the ANM-based and Ori-based PROTAC systems also include homo-PROTACs. Initially, we repurposed AS1411 to create homoAS1411, where two AS1411 molecules are linked by a six A-T base pair linker, mediating the self-degradation of MDM2 [173]. Compared to Nutlin-3 derivative-based homo-PROTACs, homoAS1411 exhibited superior degradation potency, with DC_50_ values of ~ 1 μM and ~ 71 nM, respectively [164, 173]. We also assessed the sustainability of this PROTAC by analyzing NCL protein levels in response to varying homoAS1411 concentrations. A decline in NCL levels was observed only above 2500 nM. This ubiquitination preference may be caused by the acetylated state of NCL in proliferating cells, as its acetylation and ubiquitination compete for the same lysine residue [175]. After optimizing the linker length, we demonstrated that the elongated molecule exhibited greater degradation potency. This improvement may be attributed to the linker's ability to facilitate complex formation by overcoming steric hindrance [173]. Interestingly, Ori-based homo-PROTACs not only facilitate the degradation of MDM2 but also its homolog MDMX, which lacks E3 ligase activity [175]. These homo-PROTACs promote the homodimerization and heterodimerization of MDM2/X to induce their degradation through the molecular bridge NCL-PROTAC-NCL. This dual degradation mechanism provides a more robust antitumor effect by simultaneously targeting both oncoproteins. Consistent with homoAS1411, Ori-based homo-PROTACs only facilitate NCL degradation at over 2000 nM [175]. The preferential degradation of MDM2/X over NCL might be caused by spatial arrangement, which warrants further structural biology studies for confirmation.

## Conclusions and perspectives

The landscape of drug discovery has been significantly impacted by the challenges associated with targeting "undruggable" proteins, which are typically characterized by the absence of suitable binding sites for small molecules or involvement in protein–protein interactions that defy conventional inhibition strategies. This limitation restrains the development of effective therapies, particularly in oncology where drug resistance is a pervasive issue that diminishes the long-term efficacy of treatments [23]. Targeted Protein Degradation, particularly through PROTACs, offers a promising solution by leveraging the cell's natural proteostasis network to degrade target proteins, rather than merely inhibiting them [35]. Notably, PROTACs require only transient binding to the target protein to initiate degradation, making them particularly well-suited for targeting undruggable proteins [81].

CRBN and VHL are the most widely used E3 ligases in PROTAC design due to their well-characterized mechanisms and high specificity of ligands [81]. These ligases have been successfully employed to degrade a variety of proteins, demonstrating the versatility of PROTAC technology. While CRBN and VHL dominate the current landscape, MDM2 is emerging as a promising alternative E3 ligase for PROTAC development [159]. With the dual functionality of MDM2, researchers have designed MDM2-harnessing and MDM2-targeted PROTACs to mediate specific protein degradation for therapeutic application [50, 54, 159]. In our previous research, we made a head-to-head comparative analysis of the efficacy of a CRBN-based PROTAC (dBET1) and an MDM2-harnessing degrader (Ori-JQ1-1) in degrading BRD4 [175]. Despite dBET1's lower molecular weight (785.3 g/mol) compared to Ori-JQ1-1 (874.6 g/mol), we observed that Ori-JQ1-1 exhibited superior degradation efficiency and therapeutic efficacy at equivalent doses [175, 202]. We propose that the enhanced efficacy of Ori-JQ1-1 relative to dBET1 arises from two key factors. On the one hand, the NCL-dependent internalization mechanism of Ori-JQ1-1 significantly boosts lesion-specific targeting and cellular permeability, ensuring more efficient delivery to its intended site of action. On the other hand, the difference in efficacy may also be attributed to variations in CRBN and MDM2 abundance across cell lines [175]. Regarding oral bioavailability potential, direct head-to-head comparative oral bioavailability data for these specific degraders were not included in our previous study [175], and further investigation is warranted. Notably, in accordance with established oral PROTAC design principles (ARV-110 and ARV-471), optimizing both cellular permeability and metabolic stability represents a critical determinant of candidate efficacy and oral bioavailability [203]. To achieve these dual objectives, multiple SAR strategies are routinely employed, such as systematic modulation of lipophilicity, strategic introduction of metabolic protective groups, and three-dimensional conformation control through linker rigidification and hydrogen-bond donor masking [204–207].

MDM2-harnessing PROTACs offer several potential advantages over CRBN- or VHL-based PROTACs. First, they exhibit enhanced tumor selectivity due to MDM2’s frequent overexpression in p53-wild-type cancers, potentially reducing “on-target, off-tumor” toxicity associated with the ubiquitous expression of CRBN and VHL [208]. Second, MDM2-harnessing PROTACs may circumvent common resistance mechanisms observed with CRBN/VHL degraders, such as CRBN loss in multiple myeloma [209]. Third, MDM2 demonstrates broader ligand-binding diversity, tolerating chemotypes like cis-imidazoline, spiro-oxindole, and benzodiazepinedione scaffolds. In contrast, CRBN binding is restricted to ligands containing an imidazolidine-2,4-dione core, while VHL necessitates a hydroxyproline residue [95, 210, 211]. Additionally, MDM2-harnessing PROTACs can simultaneously degrade oncoproteins and reactivate p53 via MDM2 inhibition, enabling dual mechanisms of action [54]. Whereas CRBN degraders are limited by immunomodulatory toxicity and VHL degraders by hypoxia-dependent inefficacy, MDM2-targeted degraders potentially offer a superior tumor-specific safety profile [103, 212]. Further optimization is needed to address p53-mutant tumors and MDM2-related on-target toxicities, but MDM2-harnessing PROTACs represent a promising alternative or complementary strategy to conventional E3 ligase-utilizing PROTACs.

Since many MDM2 inhibitors work by disrupting the MDM2-p53 interaction, leading to p53 stabilization and activation, this activation triggers cell cycle arrest, senescence, or apoptosis, and tissues with high turnover rates are most affected, causing widespread"on-target, off-tumor"toxicity [213]. Although the negative feedback loop of p53 and MDM2 exists, this initial, unavoidable p53 activation causes significant on-target toxicity before the feedback loop can suppress it [151]. In the clinical trials of MDM2 inhibitors, hematological toxicity, such as neutropenia and thrombocytopenia, is frequently observed as dose-limiting adverse events [214]. This phenomenon correlates with the physiological role of MDM2 in maintaining hematopoietic homeostasis through regulation of p53 stability and transcriptional activity [215, 216]. Notably, bone marrow cells exhibit higher MDM2 expression levels compared to other normal tissues, a feature that may predispose these cells to preferential inhibitor uptake, thereby disrupting hematopoiesis [216]. However, clinical trials of the dual MDMX/MDM2 inhibitor ALRN-6924 demonstrated reduced myelosuppressive effects in treated cohorts. Emerging evidence suggests that this differential toxicity profile may arise from the dual target of both MDMX and MDM2 and favorable PK properties, which collectively mitigate on-target hematological toxicity [217].

The inherent negative feedback loop between p53 and MDM2 presents a fundamental challenge for most of the MDM2-p53 interaction inhibitors [151]. This rapid compensatory MDM2 upregulation severely limits the duration and intensity of the therapeutic p53 response, promotes resistance mechanisms, and significantly constrains efficacy. Furthermore, managing the resulting on-target toxicities in normal tissues necessitates intermittent dosing schedules, complicating treatment regimens. Overcoming this self-defeating feedback loop remains a major hurdle in developing clinically viable MDM2 inhibitors, driving the urgent need for novel therapeutic strategies. Unlike traditional inhibitors, KT-253 utilizes a PROTAC mechanism to catalytically degrade MDM2 protein itself [200]. Early clinical reports from its phase 1 trial in advanced lymphoma/ALL patients indicate a notably different safety profile with no observed neutropenia or thrombocytopenia, the dose-limiting toxicities typically associated with MDM2 inhibitors [200]. This preliminary data suggests that degrading MDM2, rather than inhibiting its binding, may circumvent the feedback-driven toxicity and efficacy limitations. Moreover, new E3 ligase recruitment strategies might also avoid such toxicities, instead of utilizing MDM2 ligands that block MDM2-p53 interaction. Our group has developed NCL-bridged PROTACs, leveraging the natural MDM2-NCL binding [172–175]. This indirect recruitment of MDM2 effectively maintains the interaction between p53 and MDM2, without causing p53 hyperactivity [175]. The bridged PROTACs strategy attracts researchers’ attention and broadens the toolkit of PROTACs design.

The development of MDM2-harnessing and MDM2-targeted PROTACs represents a significant advancement in targeted protein degradation. The specific features and versatility of MDM2 provide convenience for PROTAC development and exhibit a unique therapeutic modality. Meanwhile, the progression of MDM2-harnessing and MDM2-targeted PROTACs offers tantalizing insights into broader PROTAC molecular design and optimization.

## Data Availability

No datasets were generated or analysed during the current study.

## Abbreviations

TPD: Targeted protein degradation
PROTACs: Proteolysis-targeting chimeras
MDM2: Mouse double minute 2
POI: Protein of interest
UPS: Ubiquitin–proteasome system
ALP: Autophagy-lysosome pathway
PTMs: Post-translational modifications
CMA: Chaperone-mediated autophagy
LYTACs: Lysosome-targeting chimeras
AbTACs: Antibody-based PROTACs
ATTECs: Autophagosome-tethering compounds
AUTOTACs: Autophagy-targeting chimeras
HyTTDs: Hydrophobic tag tethering degraders
CRBN: Cereblon
VHL: Von Hippel-Lindau
AR: Androgen receptor
EGFR: Epidermal growth factor receptor
CDKs: Cyclin-dependent kinases
PARP1: Poly (ADP-Ribose) Polymerase 1
TKIs: Tyrosine kinase inhibitors
RTKs: Receptor tyrosine kinases
SMARCA2: SWI/SNF-related BAF chromatin remodeling complex subunit ATPase 2
HBDs: Hydrogen bond donors
bRo5: Beyond Rule of 5
PK: Pharmacokinetic
SAR: Structure–activity relationship
BRD4: Bromodomain-containing 4
cIAP: Cell inhibitor of apoptosis proteins
SCF: Skp1-Cullin-F-box
MetAP-2: Methionine aminopeptidase-2
HIF-1α: Hypoxia-inducible-factor-1 α
PEG: Polyethylene glycol
SNIPPER: Specific and non-genetic IAP-dependent Protein Eraser
CRABP-I/II: Cellular retinoic acid binding proteins I and II
BTK: Bruton’s tyrosine kinase
NLS: Nuclear localization signal
NES: Nuclear export signal
ARF: ADP-ribosylation factor
NoLS: Nucleolar localization signal
Rb: Retinoblastoma
SUMO: Small ubiquitin-like modifier
CRM1: Chromosomal region maintenance 1
EMT: Epithelial-to-mesenchymal transition
IGF-1R: Insulin-like growth factor 1 receptor
DSBs: DNA double-strand breaks
MRN complex: MRE11-RAD50-NBS1 complex
ATM kinase: Ataxia Telangiectasia Mutated kinase
DBD: DNA-binding domain
CRPC: Castration-resistant prostate cancer
DSARTC: Double-stapled AR targeting peptide PROTAC
T1DM: Type 1 diabetes mellitus
T2DM: Type 2 diabetes mellitus
IRS-2: Insulin receptor substrate-2
NCL: Nucleolin
G4: G-quadruplex
ANM: AS1411-NCL-MDM2
ODNs: Oligodeoxynucleotides
lncRNA: Long non-coding RNA
Ori: Oridonin
UA: Ursolic acid
GAA: Ganoderic acid A
AP-1: Activator protein 1
NF-κB: Nuclear transcription factor-κB
GSPT1: G1 to S phase transition 1
ALL: Acute lymphoblastic leukemia
AML: Acute myeloid leukemia
